# BABEL: using deep learning to translate between single-cell datasets

**DOI:** 10.1038/s42003-021-02135-9

**Published:** 2021-05-13

**Authors:** George Andrew S. Inglis

**Affiliations:** Communications Biology, https://www.nature.com/commsbio/

## Abstract

Recent advances in sequencing and barcoding technologies have enabled researchers to simultaneously profile gene expression, chromatin accessibility, and/or protein levels in single cells. However, these multiomic techniques often pose technical and financial barriers that limit their practicality. Kevin Wu and colleagues recently developed BABEL, a deep learning algorithm that can effectively translate between transcriptomic and chromatin profiles in single cells, thereby enabling researchers to perform multiomic analyses from an individual dataset.

**Figure:**
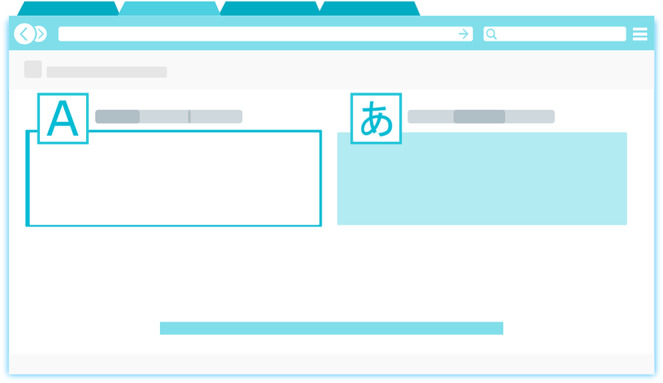
Pixabay

Technological advances within the past decade have allowed researchers to generate multiomic profiles within single-cells: gene expression and chromatin accessibility (SNARE-seq, sci-CAR), gene expression and protein epitopes (CITE-seq), or chromatin accessibility and protein epitopes (Pi-ATAC, ASAP-seq). While these methods enable multimodal analyses of gene regulation with single-cell resolution, the protocols are both technically challenging and expensive, potentially restricting their feasibility among researchers.

As an alternative approach to these multiomic methods, Wu et al.^1^ at Stanford University recently developed BABEL, a deep learning tool that can effectively translate one single-cell modality (chromatin accessibility) into a paired dataset (gene expression) for downstream analysis. The authors first trained BABEL on three cell types that had been separately profiled using single-cell (sc)ATAC-seq (chromatin accessibility) or scRNA-seq. They reported that BABEL was capable of using scATAC-seq data to robustly predict gene expression, maintaining a strong correlation with complementary scRNA-seq datasets and outperforming tools like ArchR or MAESTRO that impute gene activity from chromatin accessibility. Furthermore, BABEL-derived gene expression data preserved similar clustering and cell type-specific expression patterns observed in complementary scRNA-seq datasets. The authors also observed that BABEL could infer gene expression from scATAC-seq data in multiple human samples that were not used when training the algorithm, such as lymphoblastoma cell lines or clinical basal cell carcinoma isolates. Interestingly, BABEL also maintained a high level of accuracy when analyzing cerebral cortex or skin samples taken from adult mice, suggesting that it may be applicable for single-cell analyses across multiple species. While the authors primarily focused on translating human scATAC-seq results into analogous gene expression data, they also demonstrated that BABEL could work in reverse, inferring chromatin accessibility from scRNA-seq data. Similarly, BABEL could translate scATAC-seq input into protein epitope expression, highlighting its flexible framework.

Altogether, BABEL offers a robust and versatile approach to translating single-cell datasets between modalities. Given the practical and financial barriers to performing multiple single-cell experiments, BABEL represents a promising tool to make multimodal analyses more accessible to researchers and thereby maximize the potential of their data.
